# Understandings of disease among Pacific peoples with diabetes and end‐stage renal disease in New Zealand

**DOI:** 10.1111/hex.12946

**Published:** 2019-08-01

**Authors:** Jacqueline Schmidt‐Busby, Janine Wiles, Daniel Exeter, Timothy Kenealy

**Affiliations:** ^1^ Counties Manukau Health Middlemore Hospital Auckland New Zealand; ^2^ Faculty of Medical and Health Sciences, School of Population Health University of Auckland Auckland New Zealand; ^3^ Faculty of Medical and Health Sciences, School of Medicine University of Auckland Auckland New Zealand

**Keywords:** diabetes, end‐stage renal disease, illness representations, multigenerational legacy of diabetes, pacific peoples, self‐management

## Abstract

**Background:**

Compared with New Zealand Europeans, Pacific peoples in New Zealand develop type 2 diabetes at a higher rate and a younger age, and have 3.8 times higher incidence of end‐stage renal disease (ESRD).

**Objective:**

To investigate contextual factors that shape understandings of disease for Pacific peoples with diabetes and ESRD.

**Methods:**

Focussed ethnography. In‐depth interviews were conducted with 16 Pacific people on haemodialysis for diabetic ESRD, in Auckland, New Zealand. Study participants aged between 30 and 69 years old were of Samoan, Cook Islander, Tongan, Niuean or Tokelauan ethnicity. Thematic analysis was used to code and identify themes.

**Results:**

Participants were embedded in a multigenerational legacy of diabetes. The limited diabetes‐related education of earlier generations influenced how future generations behaved and understood diabetes. Perceptions were compounded by additional factors including the invisibility of early‐stage diabetes; misunderstandings of health risks during communication with health providers; and misunderstandings of multiple conditions’ symptoms and management. Participants had limited engagement with health services until their diagnosis of ESRD acted as a trigger to change this behaviour. However, this trigger was not effective in itself—rather, it was in combination with relevant education delivered in a way that made sense to participants, given their current understandings.

**Conclusions:**

Illness representations drive choices and behaviours with respect to self‐management of diabetes and engagement with health services. Diabetes is often present in multiple generations of Pacific people; therefore, illness representations are developed and shared within a family. Changing illness representations requires engagement with the individual within a family context.

## 1 INTRODUCTION

Compared with New Zealand Europeans, Pacific peoples in New Zealand develop type 2 diabetes mellitus (diabetes) at a higher rate^1^ and a younger age, and have a 3.8 times higher incidence of end‐stage renal disease (ESRD).^2^ More than two thirds of Pacific ESRD is due to diabetes compared with just more than one third in New Zealand Europeans.^2^ Furthermore, diabetes and ESRD are increasingly being seen across multiple successive generations of Pacific families. Our findings demonstrate how understandings of diabetes are shaped by multigenerational family beliefs and behaviours and are further compounded by misunderstandings and limitations to engagement with health providers.

Control of type 2 diabetes, sufficient to reduce complications, requires constant self‐management which becomes more complex in the presence of complications and comorbidities. Effective self‐management requires health literacy and self‐management skills supported by patient‐provider communication, which, to be effective, must respond to culture‐specific understandings of diabetes.^3^

As a key determinant of health,^4^, ^5^ health literacy is critical to personal empowerment. In its broadest sense, health literacy is a ‘composite’ term that can include ‘knowledge’ and ‘understanding’ as targets of health promotion activities.^4^ Health care is complex and health literacy is best defined in terms of what it enables us to do,^6^ noting that people with adequate general literacy may not necessarily have adequate health literacy.^7^ Furthermore, health literacy is constructed within a social context—in environments that include families, communities and social networks.^4^, ^8^, ^9^

Family context is pivotal to an individual's understanding, beliefs and expectations (illness representations) of diabetes.^10^, ^11^ Because diabetes is epidemic, and commonly present across multiple family generations, judgements, (biased) perceptions and myths associated with disease can develop within families.^11^ What is observed within the family becomes the ‘appropriate’ or normalized way to care for, or self‐manage, diabetes; thus, the platform is established for an individual's foundational knowledge and illness representations^12^ about diabetes.

Diabetes is largely asymptomatic in the years preceding a diagnosis or even to the onset of complications. This invisibility can lessen an individual's motivation to engage in diabetes self‐management. The difference between an individual's illness representations^10^, ^11^, ^12^ and a clinical assessment can strongly influence adherence or commitment to recommended treatments.^13^, ^14^, ^15^ Leventhal and colleagues propose a model of beliefs in five domains to conceptualize how illness representations determine judgement of illness and health behaviour.^12^ These beliefs address the label or diagnosis of diabetes and its associated symptoms (identity); possible causes of diabetes (causes); duration of diabetes (timelines); perceived physical, psychological or financial consequences of diabetes (consequences); and feelings of self‐efficacy for managing illness (controllability). Perceptions of consequences and controllability^14^ are prominent within the literature of diabetes outcomes where increasing evidence associates a family history of diabetes with a sense of inevitability and lack of controllability.^10^, ^14^

The traditional Pacific Island worldview of health differs from a Western or European perspective. Expressed as a notion of maintaining social order and harmony, health is viewed as a holistic concept of well‐being (a balance of the body, spirit and mind), incorporating spirituality, and values and obligations centred around family kinship and communalism.^16^ Although the importance of family among Pacific has been maintained since Western contact, there have been noticeable shifts in cultural orientation and health perceptions.^17^ Many Pacific peoples, especially migrants, have adapted their cultural beliefs about health to accommodate a biomedical Western paradigm; most likely because of their interactions with Western health services.

Within the Pacific construct of health, illness is seen as disharmony and imbalance. Common beliefs within this worldview maintain that there were less illness and disease in the Pacific prior to Western contact, and that illnesses experienced post‐contact are Western in origin.^16^ Consequentially, distinctions have been made between what are conceptualized as Pacific illnesses and those ascribed as Western illnesses, and two different systems of treatment perceived as needed.

Compared with the Western world, in the Pacific Islands new technologies, consumption of non‐traditional foods and increased international social contact appeared within a relatively short period of time. Similarly, the epidemiological transition to diabetes was rapid, especially in the Polynesian Pacific Islands.^18^, ^19^ Diabetes management in Pacific Island nations has been inhibited by social, political and economic structures that prevent or limit access to medicines, together with insufficient numbers of adequately trained health workers and health educators.^20^

Concerns about general Pacific health and well‐being in New Zealand are not new.^21^, ^22^, ^23^, ^24^ National prevention strategies have identified disparities in access, provision and coordination of health services,^25^, ^26^ with government and sector reports proposing interventions to improve health outcomes for Pacific peoples.^27^, ^28^, ^29^ A low degree of engagement with health services by Pacific peoples has been identified as a central reason underpinning their much poorer health status compared with the general New Zealand population.^27^ Reasons for low engagement include issues relating to communication, lack of trust and experiences of discrimination influencing decisions to engage with services and low health literacy.^15^, ^27^, ^29^, ^30^

For these reasons, commentators suggest that there is a need to determine baseline levels of knowledge and understanding of diabetes within Pacific Island communities so that effective strategies for patient engagement can be developed.^27^ However, research relating to low health literacy often centres upon *‘what* is understood’ rather than ‘*what* are the underlying contexts and constructs explaining *why* things are understood in that way’.^9^ The research reported here forms part of a larger focused ethnographic study that sought to address the question ‘what is the lived reality of illness for Pacific peoples with diabetes?’ In this article, we investigated the contextual factors that shape understandings of disease for Pacific peoples with diabetes and ESRD.

## 2 METHODS

### 2.1 Design, setting and sampling

This qualitative enquiry employed a focused ethnographic approach, which, unlike traditional ethnography, examines a single issue or experience of a subgroup of people within a context‐specific setting.^31^ The larger study aimed to understand the contexts and meanings underpinning self‐management behaviour—through participant accounts of living with diabetes. As an applied research method, focused ethnographic approaches facilitate better understandings of complexities surrounding an issue or phenomenon from the perspective of the participant.^32^ In this context, the researcher can examine personal and societal factors that influence, for example, decision making. Cruz and Higginbottom^33^ among others reinforce this argument, asserting focussed ethnographies enable the researchers to consider how people integrate health beliefs and practices into everyday life.^34^

A purposive sampling strategy (seeking maximum variation) was used to identify patient‐participants from two in‐centre haemodialysis units (facilities reserved for those who are most dependent and most unwell) within a large District Health Boards (DHB) in New Zealand, serving a high Pacific Island population. The inclusion criteria for the study comprised the five most common Pacific ethnicities in New Zealand (Samoan, Cook Islander, Tongan, Niuean or Tokelauan), individuals aged 20 years or more who had type 2 diabetes and ESRD (undergoing dialysis); lived within the DHB catchment area; and had a good command of the English language. The sample was restricted to those with type 2 diabetes and ESRD, because we believe those whose health has progressed to an endpoint in which life is now time‐limited, may provide a deeper insight into the reality of diabetes risks and consequences along with lessons learned—albeit too late for them personally. Participants with a ‘good command of English’ were selected because the researchers believed the original meanings within participant responses would be retained and not be lost during translation between languages. Study information packs were made accessible in general spaces within the units for prospective patients to read and self‐nominate if they wished to take part in the study.

### 2.2 Data collection

Data were collected by the first author (JS‐B) who made field observations, conducted face‐to‐face interviews and recorded field notes. Interviews were between 30 and 80 minutes, in English, and undertaken while participants were undergoing haemodialysis and were either lying on their bed or sitting semi‐recumbent in a chair. The interviewer stopped the shortest interview after noticing the participant was becoming increasingly breathless. Three interviews were re‐scheduled due to general unwellness, despite participants in each case wanting to tell their story. Those three interviews were conducted at their next dialysis treatment. Another three participants wanted to be interviewed during the initial recruitment meeting ‘*because I might not be here next week*’ (Sa.2). A relaxed conversational approach easily accommodated interruptions from nurses who attended to alarms or checked monitor readings. The interview aimed to explore: understandings of diabetes and kidney disease; health information and communication; self‐management; and reflections on diabetes (Table 1). Written informed consent was gained prior to interview, and all participants consented to their interview being audio‐recorded. Interviews were transcribed, and interview data were managed using Nvivo12‐Pro and Microsoft Excel.

**Table 1:** Interview topic guide

Personal background: NZ/Island born; Additional conditions (self; family); Hospital admissions	
Diabetes: *…Thinking back about having diabetes…* Diabetic kidney disease: *…When you found out your kidneys were not working too well…* ESRD: *…Now that you're on dialysis…*	Thoughts and feelings; expectation of disease Perceptions or understandings of ESRD; self‐management (incl. diabetes) Symptom interpretation and management Information received/education received (incl. health provider interactions)
Comorbidities: Knowledge of and in relation to; Diabetes; kidney disease; ESRD	
Knowing what you know *now* about diabetes and kidney failure: Reflections? Things you would do differently?	
Messages to family/ others/ health providers Words of wisdom? How explicit do the messages need to be?	
Other comments	

### 2.3 Data analysis

A thematic analysis approach informed by Saldaña^35^ was used to identify contextual factors underpinning participants’ understandings of diabetes and ESRD. All identifying information on transcripts were anonymized prior to being read by those other than the interviewer. Transcripts were read several times by members of the wider research team. Data were categorized using first cycle coding (descriptive or basic labelling) prior to being reorganized into groups based on shared characteristics using second cycle coding (pattern coding).^35^ Initial themes were then refined into confirmed themes addressing the research question. The authors represent the disciplines of health services (JS‐B); health and medical geography (JW; DE); and medicine (TK). JS‐B is also Samoan. Advisors additional to the research team represented sociology, diabetes nursing and Pacific health, and supported coding and interpretation.

The study was guided by our obligation to conduct trustworthy research throughout the enquiry.^36^ As an important component of trustworthiness, credibility was supported by prolonged engagement, peer debriefing, insider knowledge, crystallization and reflexivity. Field observations were also recorded along with reflective notes and memos to minimize distortion of participant information.^37^ Furthermore, to enable participants experiences to become meaningful and resonant to the reader,^38^ rich descriptive accounts of experiences and the context within which they occurred are provided.

## 3 RESULTS

Of the 42 potential participants who indicated an interest in the study, 9 did not meet the inclusion criteria and 17 withdrew due to hospitalization (2), feeling unwell (4) and a change of mind (11). Sixteen took part in the study comprising 5 Pacific ethnicities: Cook Islander (6), Samoan (5), Tongan (3), Niuean (1) and Tokelauan (1). Thirteen were Island‐born. All were aged between 37 and 68 years, and half were female. Nine had three or more comorbidities in addition to ESRD (Table 2).

**Table 2:** Participant characteristics

Code	Age group	NZ born	Time in NZ (years)	Length of illness (estimated years)		Comorbidities
				DM	ESRD	
Ck.1	50‐59	No	43	37	5	DF; CVD
Ck.2	40‐49	No	21	10	1	ED; G; CVD; LD
Ck.3	50‐59	No	41	30	9	ED; CVD; HTN; O(4)
Ck.4	50‐59	No	50	20	1	G; LD; O(1)
Ck.5	60‐69	No	54	38	3	G, LLA; O(2)
Ck.6	50‐59	No	45	13	<1	HTN
Ni.1	60‐69	No	38	27	17	CVD; HTN;
Sa.1	40‐49	Yes	>40	10	10	A; DF; G; CVD; HTN; LD
Sa.2	60‐69	No	48	32	13	CVD; HTN
Sa.3	40‐49	No	29	19	3	A; ED; HTN; LD; O(1)
Sa.4	40‐49	Yes	>40	15	5	CVD; HTN,
Sa.5	30‐39	Yes	>30	9	2	HTN; O(2)
Tg.1	30‐39	No	38	18	2	ED; DF; G; CVD; LLA; O(2)
Tg.2	30‐39	No	7	10	1	ED; HTN
Tg.3	60‐69	No	38	16	3	O(2)
Tk.1	50‐59	No	52	20	3	HTN; O(2)

Comorbidities: A asthma; DF diabetic foot; ED eye disease/cataracts; CVD cardiovascular disease; G gout; HTN hypertension; LLA lower limb amputation; LD liver disease; O other conditions.

The results are summarized under two central themes: (a) understandings of diabetes, which describes how participants’ perceptions of diabetes were formed and how these perceptions influenced self‐management and decision making about health prior to diagnosis with ESRD; and (b) speaking from experience, which describes participants’ hindsight about their own lessons learned and what they believe may help others with, or at risk of, diabetes and other chronic diseases.

### 3.1 Understandings of diabetes

#### 3.1.1 Diabetes, the name of a sickness adults get

All participants reported diabetes was a common condition within their families and most could recall parents, grandparents and other family members with diabetes over several generations. They had heard of *diabetes* growing up but could not recall conversations about the disease and were not aware of serious complications; *‘it was just the name of a sickness the adults get*’ (Sa.3).

I’m not really understand[ing]. The diabetes is in my family, yes. But that’s all I knew back then. My mother got, my father got it, yeah. They didn’t talk to us childrens (sic) about it. I know it now, it’s bad, very, very bad. (Ck.1)

I used to watch my grandparents prick their fingers and I used to see them taking tablets and I never understood what it was until I got it. (Sa.4)

Commonly within Pacific cultures, there are conversations that only the adults engage in, and it was usual and expected that children were not to ask questions. In hindsight however, this participant and others felt strongly that parents had a responsibility to talk more openly about health conditions. Many participants stated they now doubted that their parents and grandparents had fully understood diabetes, its complications or appropriate management, and thought this might also have been why parents and grandparents had appeared not to worry about the condition or discuss it in family conversations:

My parents and them [grandparents] didn’t really say much about these things either. It was like having a cold or a sprained ankle, they got sick and then got over it. Although now, I know the sick is always there….They just seemed to go with the flow…No one seemed distressed or you know, as if it was a major or nothing, yeah, just like having a cold. (Tk.1)

My Mum and Dad they didn’t speak about [it] to us. I don’t know if they really knew back then, you know. All the information’s available now…I only know [about] then ‐ from what they know and what I seen. But now, I know heaps more about it. (Ck.3)

#### 3.1.2 Symptoms to diagnoses

Prior to diagnosis of diabetes, most described having swollen legs and feet, headaches, sticky eyes, itchy skin, lethargy, dizziness and fainting spells. Participants recalled being given advice about high blood glucose levels and being informed of the importance of on‐going blood tests. They also remembered being told about the need to reduce eating unhealthy foods and drinks. Despite being told these things, most could not remember being told of how important these things were in relation to preventing the development of diabetes:

I didn’t know it was that bad. (Tg.1)

The doctor used to tell me how much my blood number was but I didn’t know what it was for….He told me all the time to cut down the fizzy drinks and my smoking…He never say it was serious. (Ck.3)

Only one person reported receiving a diagnosis of diabetes at an early stage of the disease. The remainder said they were told of their diabetes well after onset and most described finding out during a hospital admission or medical appointments that were perceived to be for another reason:

I had a, a cut on my leg, and it didn’t heal after about 3 months. And then I went to see the doctor, and they sent me to a lab to do some blood test and all that? So then a week, the doctor rang me back he told me I had type 2 diabetes. (Ck.2)

I had a boil on my chest…I came to see my doctor, they took some blood, and then I went to hospital and operate on that, and that’s how I found out. (Sa.3)

Three participants expected that they would develop the condition because it was ‘in their family’: *‘I was waiting for it. My parents to me and one brother*’ (Tg.2). But for the majority, although diabetes was also in their families, the diagnosis came as a shock: ‘*No [I wasn't expecting diabetes] ‘cos I don't take sugar, I don't eat sweets*’ (Ck.6).

Between diagnoses of diabetes and ESRD, information given by health providers was again often misunderstood. Participants did not always perceive their symptoms were caused by diabetes because they had other comorbidities: ‘*I don't know if my pain is for the diabetes or the other ones I’m also have it’* (Ck.1). Similar to diabetes, the realization of having ESRD was a mixture of emotions and disbelief at what could have been preventable:

I didn’t really thought it was the end of the road, it was like ‘what?’ I wasn’t sure if I was angry or sad like someone had just died…I had all the advice given to me but because I didn’t understand it, it just hasn’t sunk in and now it’s too late. (Tk.1)

Almost all participants were diagnosed with ESRD while attending health services for other issues:

…last year I got bad pneumonia, and that’s when I admitted at [hospital], the doctor say there was damage ‐ fail my kidney. (Tg.2)

I had a cyst that was growing and they removed that…I was in hospital, I was here for I think about two months…and then the dialysis nurse came and told me I needed to be on dialysis cos my kidneys were failing. (Sa.5)

##### Health was not a central priority

Participants described busy lives where health was not a central priority. Self‐management activities, such as increasing physical activity, did not always fit in with their everyday lives. One participant said she was only just coping with the stress in her life and could not manage her medications as well. Clinical appointments were frustrating with clinicians making judgements that were not seen as helpful or relevant. Practical support was rarely given:

I didn’t really kept up with my appointments for diabetic. I stopped because every time I go…they talk to me to stop this, stop that, stop the food, and the food I love, and the drinks, and you know I don’t really want to listen what the doctor said. He didn’t help what I can do in my house. (Sa.2)

…the doctor always said the same thing ‘oh it’s not good, it’s not good’. I’m ‘oh yeah ok’. Next time, ‘it’s not good, it’s not good’, me, same thing ‘oh ok, see you next time’ …Yeah he wasn’t helpful now that I’m here hooking up to this machine. (Ck.4)

I didn’t know if I’m coming or going. They tell me these things, but too many things, wanting me to go here for something and this one another thing. I had a young children to look after [more] than myself. (Ck.5)

One participant lost motivation to exercise: ‘*I was doing a lot but it still wasn't helping… like the walking was for fitness and I’m still unfit*’ (Tk.1).

The invisibility of symptoms in the early stages of diabetes also contributed to the low priority placed upon health. Many described feeling confused when attempting to understand what their doctor was telling them:

I felt ok and couldn’t see anything wrong with me each time, so just carried on as I was doing, carrying on until I finally got the message! And, got it too late!!. (Sa.5)

…I feel normal and the doctor say no you sick. Yeah that’s why I’m confused. I’m feeling normal. (Tg.3)

There were a few things now looking back that I should have picked up on. But I really didn’t understand diabetes. I didn’t even pay attention to the doctors or go to the appointments. How was I supposed to know how important it was? I felt sick then I felt ok. I thought it was ok in the beginning. (Tg.1)

### 3.2 Speaking from experience

As adults, participants witnessed the consequences of diabetes and its serious complications in close family members. Some reported seeing the same level of care:

…we understand that he’s [Dad’s] got a kidney problem but he wasn’t dying because of the kidney problem ‐ he was dying because his heart broke down. He had a leg amputated in August and he passed on September. (Sa.3)

All participants acknowledged their diabetes was not because they were Pacific. They said their current situation was because they did not understand the disease of diabetes:

No, no, there’s nothing PI [Pacific Islander] about where I am. Sure there’s a lot of us, but that’s just ‘cos we all missed the boat on this one. How do you explain the other countries getting diabetes….No, PI is not the problem, it’s my ears and my understanding of the situation. (Ck.4)

…if anything it should help us ‐ me from getting to this stage. We got enough examples out there but, yeah, no. It’s because I didn’t get the drift back then, too busy being a cool dude and thinking I’ll be ok, no worries…. (Sa.5)

The regret of not managing their diabetes was often combined with an overwhelming sadness. Although painful, these emotions were a catalyst to helping others in the family avoid getting diabetes and ESRD. Commonly, participants wanted others to be free of diabetes: *‘have no diabetes or machines or sickness’* (Ck.1). They told their stories to family in the hope that it might build a better understanding of diabetes—a condition that needs to be taken seriously:

I didn’t want him to throw away his life. He got two good kidneys but he’s coming towards this [dialysis]. ‘Uncle I’m all good, I’m just fine, I only need one kidney’ that’s all they say. I’m angry, looking at myself, I want to live. ‘Ok then [I said], give me one kidney. If you only think you need one, give me the other one!. (Ck.2)

That’s why it’s important for families to understand ‐ even though they haven’t got it [diabetes], they should still need to know what is going on… Tell them, tell them, wake up”. (Ck.1)

I’m telling my children and my grandchildren…don’t be like me now, I don’t want any of you to go on a machine like me…it’s no, you know, no life!. (Sa.2)

Participants’ conversations reflected a need for clearer messages from health providers. In reflecting upon their conversations with doctors, many were unaware of their overall health risk and so did not realize the importance or urgency of the information being given to them:

…they just kept telling me oh your kidneys are leaking… [I] never took any notice eh cos it was just like ‘oh yeah’. But it wasn’t like ‘if you don’t pay attention now to your health, this is what’s going to happen further down the track…’. (Sa.5)

One doctor said to me, I’m 30 then, ‘you know, when you’re 40 and I don’t know if you’re going to be here’ ‐ I said pardon? ‘I don’t know how long you’re going to be here with your health’…[at] 40 ‘I don’t think you’re going to be here at 50’. I’m 50 now ‐ I’m still here!. (Ck.4)

They kept telling me that my kidneys were … my numbers were low. Yeah I didn’t understand about numbers. I just thought it would come right if I could change my diet and stuff like that…and I didn’t ask either. (Tk.1)

When comparing previous education sessions to education received since being diagnosed with ESRD, all participants agreed on how useful the information and education would have been before or at the time of being diagnosed with diabetes. Many told of how they now felt more confident and knowledgeable about diabetes and looking after themselves. They had learned about how dialysis works, caring for their fistula or graft, common side‐effects related to dialysis treatments and self‐managing ESRD including diet, fluid intake and general lifestyle advice. Participants reported education felt personalized for their own conditions unlike previous sessions for diabetes that was in a classroom and generalized:

I ask what I want to ask…The nurse she explain to me the things I need for my health, my diabetes, my foot, my kidney. (Ck.1)

Yeah they told me about it but it was more just giving information…all about diabetes, not ‘me with the diabetes’…But when I started this I’ve had so much help, like dieticians, the nurses telling us, educating us. If we can prevent that even before we come on this, if we had the same help before like around the diabetes and stuff, I think I wouldn’t even be on this. (Sa.5)

When asked about what might prevent future generations developing diabetes and ESRD, the majority advocated towards including more education about the interactions and management of multiple‐comorbidities, and using different modes of delivery:

To learn about the different diseases eh, how diabetes affects the heart and the heart blood pressure the diabetes and my legs. These kids don’t know those things. (Ck.6)

Sometimes people need to experience the sickness, like we do when the people died, they body is dead, but it’s real eh? Yeah, the same thing to sickness. I come here and you know the smell, sometimes it’s worse for the leg cut off but not healing, aawww I know it!. (Ni.1)

## 4 DISCUSSION

These findings explore the experiences of living with diabetes, and its progression to ESRD, for 16 Pacific Island adults in New Zealand. Irrespective of age, gender or ethnicity, participants recounted similar experiences in relation to their knowledge of diabetes, diabetes self‐management and illness representations within their families. The diagnosis of ESRD created a disruption that impacted them emotionally, physically and socially. Although they accepted this reality, participants blamed themselves for making bad decisions about health care and were angry that they had missed earlier opportunities to manage their diabetes.

The literature repeatedly confirms that health‐care decision making is influenced by an individual's perception(s) of their control over diabetes and the severity of consequences related to becoming ill.^10^, ^15^, ^39^ Participants made sense of their diabetes in the context of their own lives. Childhood memories of family members with diabetes influenced how participants understood diabetes as adults. Understandings of health and illness had largely been shaped by family illness representations over generations.^14^, ^15^, ^39^ These family perceptions and misconceptions of diabetes have faced limited challenge and re‐direction because of a long‐standing shortage of health educators in the Pacific across time.^20^ Scollan‐Koliopoulos and colleagues suggest family are the first diabetes educator where an individual's understanding of diabetes is learned by witnessing role‐modelled beliefs and behaviours.^10^ These beliefs and behaviours became deeply rooted within families and the knowledge they collectively share. These authors further assert that common understandings shared in past and current generations will most likely be positioned similarly in future generations. We note the difficulty of shifting entrenched beliefs and understandings, described in the decision‐making literature as anchoring and adjustment error, where new information only partially ‘adjusts’ beliefs away from where they are ‘anchored’.^40^

According to Leventhal's model of illness representation, individuals construct a representation of reality from perceptions within their environment.^12^ For Pacific peoples, family is central to the conception of health^16^ and as shown in this study, family does shape an understanding about illnesses, such as diabetes. The experiences and insights learned from previous generations proved insufficient for participants to self‐manage their diabetes, and participant narratives suggest they generally experienced more comorbid conditions than previous generations. Their illness perceptions were not well informed because families were not well informed. This further suggests early interventions were not accessed and contributed to diabetes often being diagnosed at an advanced stage. In hindsight, participants questioned whether previous generations had sufficient knowledge of diabetes.

Participants reported their understanding of diabetes changed following dialysis and ESRD‐related education when they believed they developed sufficient knowledge to build on what they already knew. They described realizing the importance of adopting health‐seeking behaviours, being able to ask important questions and being more confident to discuss diabetes with others.

The diagnosis of ESRD devastated participants. One person described the complex emotions and disbelief experienced in coming to terms with an incurable illness that might have been prevented at an earlier stage. The diagnosis was the trigger that engaged them in managing their conditions and compelled participants to strengthen their knowledge.

Relevant knowledge shifted participants from responding to acute symptoms to managing diabetes in the longer‐term. This shift was similarly seen in a New Zealand study of Pacific adults with chronic obstructive pulmonary disease (COPD) who also initially focused on present symptoms rather than longer‐term strategies.^41^ However, unlike the early stages of diabetes, the early symptoms of COPD such as shortness of breath and production of sputum are more obvious and persistent. For diabetes, the invisibility of symptoms can often lead to unpredictable or no response from the individual and therefore contribute to progressive complications and disabilities.^42^

A growing literature supports the significant potential contribution of health literacy to reducing the burden of disease.^4^, ^5^, ^43^, ^44^ Robust evidence highlights the need for individuals to gain contextually relevant knowledge, skills and abilities to self‐manage health conditions, especially for migrant and refugee populations where linguistic and cultural barriers often exist.^45^ Health literacy has recently been established as ‘*an identifiable and manageable risk in clinical care*’,^9^ p.3 specifically within the management of chronic and complex conditions that depend upon successful patient engagement. While, previously, health literacy education centred on the individual's skills and capabilities, the focus has shifted towards the context within which health information is obtained, understood and acted upon.^9^

The WHO argue relevant health communications must help individuals ‘*understand that there is a health risk for themselves…[and] that the risk could be severe and that they reduce that risk by undertaking recommended actions*’0.^46^ However, diabetes education programmes are often pre‐packaged to convey a general message, commonly focussing on the dominant population or group. In the current study, participants perceived these sessions as too generalized, whereas education tailored towards their own health needs was considered effective and empowering.

Messages tailored to a specific person or audience have been shown to be effective in a wide range of health outcomes including diabetes^47^ and ESRD.^48^ As a common element within health literacy models,^8^ tailoring messages can increase relevance^49^ and influence behaviour change^50^ by appealing to an individual's values, beliefs, and personal and socio‐cultural characteristics. Framing is a characteristic of health communication^49^ that enables messages to be identified and categorized within particular settings.^51^, ^52^ The way information is framed can influence decision making and behaviour.^51^ Many participants advocated messages that delivered multi‐sensory experiences such as seeing limb amputations and experiencing the smell of wounds that are not healing (Ni.1). Framing messages delivered in a factual, case‐based and persuasive manner supports a perception of credibility,^50^ enhancing consequences of diabetes risk and the consequences of impact upon an individual's life.

### 4.1 Limitations and practice implications

Participant self‐reports often covered a long period of time. Risk of recall bias was reduced through not asking for specific dates but rather asking about events in relation to one another, for example experiences *before* or *after* diagnoses of diabetes or ESRD. We do not claim participant responses are representative of all Pacific peoples. However, given the increasing global burden of non‐communicable diseases on Pacific (and migrant) populations,^53^ this study may contribute to on‐going development and risk management associated with patient engagement and disease management.

To consider family influences, we purposefully explored participant's recollections without assessing family member's views for convergence. While we believe this does not lessen the trustworthiness of our findings in respect of each participants’ reality, future research examining the perspectives of associated family members in households would add to existing understandings and literature. At an individual level, health education needs to consider how health knowledge (illness representations) is constructed and the relevancy of health information given, within the context of each individual's culture and family.

## 5 CONCLUSION

Preventing onset and/or progression of disease necessitates knowledge surrounding the consequences—the consequences of diabetes progression and the consequences to the individual's life. Messages delivering multi‐sensory experiences may prove effective in instilling a sense of realness to these consequences. Consideration of these factors can help health providers tailor messages to improve patient engagement. The framing of health messages to individuals at risk or with diagnosed diabetes requires relevancy to the individual's context within which health information is obtained, understood and acted upon.

Given diabetes is prevalent across successive generations of Pacific Island families, health providers need to consider an individual's understanding and underlying contexts for ‘*why* they are understood in that way’. This study found that family perceptions of diabetes across generations reinforced participants’ low engagement in diabetes self‐management. All 16 families experienced simultaneous diabetes across multiple generations and interviews reveal indications of ESRD following the same pattern. Findings reveal (mis)understandings about diabetes stemmed from education and communication from health providers that were insufficiently connected to the individual. If misunderstandings are embedded in the family, then efficient and effective re‐education needs to address both family perceptions and individual health needs in unison.

## CONFLICT OF INTEREST

None.

## ETHICAL APPROVAL

Institutional ethical approval was obtained from the University of Auckland Human Participants Ethics Committee: Reference 020658.

## Data Availability

The data sets generated and analysed during the current study are not publicly available as participants did not consent to transcripts of interviews being shared.
